# Anti-fibrotic effects of tannic acid through regulation of a sustained TGF-beta receptor signaling

**DOI:** 10.1186/s12931-019-1141-8

**Published:** 2019-07-29

**Authors:** Eleanor B. Reed, Shawn Ard, Jennifer La, Chan Young Park, Laura Culligan, Jeffrey J. Fredberg, Larisa V. Smolyaninova, Sergei N. Orlov, Bohao Chen, Robert Guzy, Gökhan M. Mutlu, Nickolai O. Dulin

**Affiliations:** 1 Department of Medicine, Section of Pulmonary and Critical Care Medicine, the University of Chicago, 5841 S. Maryland Ave, MC6076, Chicago, IL 60637 USA; 2 Molecular and Integrative Physiological Sciences, Harvard T.H. Chan School of Public Health, Boston, MA USA; 3 Laboratory of Biomembranes, Faculty of Biology, Lomonosov Moscow State University, Moscow, Russian Federation; 4 Siberian Medical State University, Tomsk, Russian Federation

**Keywords:** Tannic acid, Pulmonary fibrosis, TGF-beta, Smad2, Myofibroblast

## Abstract

**Background:**

Pulmonary fibrosis is a progressive disease characterized by structural distortion of the lungs. Transforming growth factor-beta (TGF-beta) is a key cytokine implicated in the pathogenesis of pulmonary fibrosis. TGF-beta-induced myofibroblast differentiation characterized by expression of smooth muscle alpha-actin and extracellular matrix proteins is a key process in pathogenesis of fibrotic disease. Tannic acid is a natural polyphenol with diverse applications. In this study, we investigated the effect of tannic acid on myofibroblast differentiation and pulmonary fibrosis in cultured cells and in bleomycin model of the disease.

**Methods:**

Primary cultured human lung fibroblasts (HLF) were used. The relative levels of proteins were determined by Western blotting. HLF contraction was measured by traction microscopy. Bleomycin-induced pulmonary fibrosis in mice was used as the disease model.

**Results:**

Tannic acid inhibited TGF-beta-induced expression of collagen-1 and smooth muscle alpha-actin (SMA) as well as force generation by HLF. Tannic acid did not affect initial phosphorylation of Smad2 in response to TGF-beta, but significantly inhibited sustained Smad2 phosphorylation, which we recently described to be critical for TGF-beta-induced myofibroblast differentiation. Accordingly, tannic acid inhibited Smad-dependent gene transcription in response to TGF-beta, as assessed using luciferase reporter for the activity of Smad-binding elements. Finally, in mouse model of bleomycin-induced pulmonary fibrosis, therapeutic application of tannic acid resulted in a significant reduction of lung fibrosis, decrease in collagen-1 content and of Smad2 phosphorylation in the lungs.

**Conclusions:**

This study demonstrates the anti-fibrotic effect of tannic acid in vitro and in vivo through a regulation of sustained Smad2 phosphorylation.

**Electronic supplementary material:**

The online version of this article (10.1186/s12931-019-1141-8) contains supplementary material, which is available to authorized users.

## Background

Idiopathic pulmonary fibrosis (IPF) is a progressive, fatal disease characterized by parenchymal fibrosis and structural distortion of the lungs. Age-adjusted mortality due to pulmonary fibrosis is increasing [1], and it poses a vexing clinical challenge given the lack of proven effective therapy. IPF is thought to be a disorder of abnormal wound healing [2, 3], wherein the initial trigger to the fibrotic response is injury to the alveolar epithelial cell, followed by an exuberant, non-resolving wound-healing response [4–6]. Injury of alveolar epithelial cells results in the elaboration of a fibrinous provisional matrix and activation of several pro-inflammatory, pro-coagulant, and pro-fibrotic mediators, of which Transforming Growth Factor-β1 (TGF-β1) is the most established [7–9]. TGF-β1 has been localized to areas of fibrosis in both experimental and human pulmonary fibrosis [7, 10, 11]. Lung-targeted overexpression of TGF-β1 results in the development of lung fibrosis in animals [9, 12]. Conversely, inhibition of TGF-β1 via soluble TGF-β receptors can inhibit in vivo fibrogenesis [13, 14].

Fibroblasts, under stimulation by TGF-β1, differentiate into myofibroblasts characterized by the de novo expression of cytoskeletal and contractile proteins, modified focal adhesion complexes [15], and components of the extracellular matrix [16–18]. Several cytoskeletal and smooth muscle proteins are expressed in myofibroblasts, including smooth muscle α-actin (SMA), the most established marker for myofibroblast differentiation [18–21]. Functionally, this phenotypic switch is thought to increase the ability of the myofibroblast to attach to the remodeling matrix and ultimately facilitate wound contraction during healing process. However, induction of the myofibroblast phenotype is also associated with an increased resistance to apoptosis [22, 23] and secretion of extracellular matrix proteins (collagen isoforms, fibronectin, etc.) and pro-fibrotic factors (connective tissue growth factor (CTGF), insulin-like growth factor (IGF-1), etc. [16, 24]), thus perpetuating ongoing tissue remodeling and fibrosis. Myofibroblasts are invariably found in histologic sections of human lung specimens from patients with pulmonary fibrosis and are thought to be a critical pathogenic mechanism responsible for the progressive nature of IPF. Disrupting cellular mechanisms responsible for the acquisition of the myofibroblast phenotype may be a potential strategy to attenuate the ongoing fibrotic response in pulmonary fibrosis.

Tannic acid is a natural polyphenol found from tara pods, gallnuts and leaves of certain plants [25]. Tannic acid has been used for chemical staining of cellulose fibers (wood, textile), as a fixative-mordant in ultrastructural microscopy studies, as aroma compound in certain drinks, and for treatment of burns in humans [26]. Recent studies have demonstrated the protective effect of tannic acid in a rat model of cardiac hypertrophy and fibrosis [27], and in a mouse model of liver fibrosis [28]. We have observed that tannic acid is a potent inhibitor of myofibroblast differentiation and sought to determine its anti-fibrotic action in vitro and in mouse model of pulmonary fibrosis, and to assess the mode of its action.

## Materials and methods

### Primary culture of human lung fibroblasts (HLF)

Human lung fibroblasts (HLF) were isolated from human lungs rejected for transplantation through the Regional Organ Bank of Illinois (ROBI)/Gift of Hope. Human lung tissue samples were placed in DMEM with antibiotics. Lung tissue was minced to ~ 1 mm^3^ pieces, washed, and plated on 10-cm plates in growth media containing DMEM supplemented with 10% FBS and antibiotics. The media was changed twice a week. After approximately 2 weeks, the explanted and amplified fibroblasts were cleared from the tissue pieces, trypsinized and further amplified as passage-1. For experiments, cells were grown in 12-well plates at a density of 1 × 10^5^ cells per well in a growth media for 24 h, starved in DMEM containing 0.1% bovine serum albumin (BSA) for 24 h, and treated with desired drugs for various times as indicated in the figure legends. Primary cultures were used from passage 3 to 9.

### Cell lysis and Western blotting

Cells were lysed in urea buffer containing 8 M deionized urea, 1% SDS, 10% glycerol, 60 mM Tris-HCl pH 6.8, 0.02% pyronin Y, and 5% β-mercaptoethanol. Lysates were sonicated for 5 s. Samples were then subjected to polyacrylamide gel electrophoresis and Western blotting with desired primary antibodies and corresponding horseradish peroxidase (HRP)-conjugated secondary antibodies, and developed by chemiluminescence reaction (Pierce). Digital chemiluminescent images below the saturation level were obtained with a LAS-4000 analyzer, and the light intensity was quantified using Multi Gauge software (Fujifilm).

### Traction microscopy

To measure contractile force of human lung fibroblasts cells, we used Fourier transform traction microscopy (FTTM) [29, 30]. The principle of this assay is to measure the deformation of a soft substrate caused by cells and to convert this measured deformation to traction (contractile force per unit area) exerted by the cells [29]. We fabricated polyacrylamide (PA) gels of which modulus is 9.6 kPa and on which 0.2 μm fluorescent beads were coated to visualize gel deformation [30]. After coating the PA gels with bovine collagen (40 μg/mL), cells were seeded on the PA gel in near confluency. Cells deform the gel by exerting traction and the deformation on PA gels resulted in the displacements of fluorescent beads. Bead displacements were measured by comparing the positions of beads before seeding cells and after the drug treatments. Based on the displacement fields, cellular traction was calculated using FTTM [29, 30]. Root mean square traction was used as an average contractile force magnitude of HLF cells [30].

### Bleomycin-induced pulmonary fibrosis

9–10 week old *C57BL/6* mice were intratracheally instilled with 1 U/kg bleomycin. Tannic acid was administered intraperitoneally daily at a dose of 10 mg/kg beginning on day-7 post-bleomycin treatment. Mice were sacrificed on day-21. Due to a patchy nature of fibrosis in this model bilateral lungs from each mouse were subjected for either histochemical, or for biochemical analysis.

### Morphological analysis of pulmonary fibrosis

Lungs were inflation fixed in 10% formalin solution in saline, all five lobes were paraffin imbedded; and lung sections were stained with haematoxylin and eosin stain (H&E). 40X images of the H&E-stained sections were obtained on a CRi Pannoramic whole slide scanner which creates an image without showing the slide name (unless inquired). The severity of fibrosis was first blindly assessed on scanned images by two investigators using modified Ashcroft scoring system [31]. Up to 150 fields at 10X magnification covering whole lobes were scored using Panoramic View program with “preview tracking history” function turned on, and the average score for each lung was calculated. The means±SD were then calculated for each experimental group. The extent of fibrosis was then assessed by measuring lung tissue surface area. Five representative 10X images from each lobe (devoid of airways or blood vessels) were captured, and the lung tissue surface area was measured using ImageJ program as follows. The total area was first quantified using the Analyze-Measure function. The white area (representing empty spaces) was then selected (with brightness set at 240) under the Image – Adjust – Color Threshold function, and was quantified using the Analyze-Measure function. The lung tissue surface area was then calculated by subtracting the white area from the total area and expressed as a fraction of the total area. Finally the histologic score of fibrosis was calculated by multiplying the Ashcroft score by fraction of the lung tissue surface area for each lung, analogous to previously described approach [32, 33]. The means±SD were then calculated for each experimental group.

For biochemical analysis, isolated lungs were rapidly homogenized for 15 s at maximum speed in 2 ml ice-cold PBS using tissue homogenizer. Homogenized samples were then mixed 1:1 (v/v) with 12 N hydrochloric acid for hydroxyproline assay, or mixed 3:1 with 4X Laemmli sample buffer, boiled for 5 min, sonicated for 5 s, and used for analysis by Western blotting.

### Hydroxyproline assay

The hydroxyproline assay was performed as described previously [34]. Briefly, lung homogenates were mixed 1:1 (v/v) in 12 N hydrochloric acid and hydrolyzed for 12 h at 110 °C. An aliquot was evaporated, resuspended in citrate-acetate buffer with chloramine T and left at room temperature for 20 min. Ehrlich’s solution was then added, and samples were heated at 65 °C for 15 min. After cooling to room temperature, absorbance was measured at 550 nm. Hydroxyproline content was determined against a standard curve generated from pure hydroxyproline.

### Luciferase assay for Smad-dependent gene transcription

Smad-dependent gene transcription was assessed using the luciferase reporter driven by four copies of Smad binding elements (SBE4-Luc) whose efficiency and selectivity for Smad activation was demonstrated previously [35]. Subconfluent cells were co-transfected with cDNA for SBE4-Luc and for a control thymidine kinase (TK) promoter-driven Renilla luciferase plasmid (pRL-TK). Cells were serum-starved, followed by stimulation with 1 ng/ml TGF-β with or without tannic acid for 48 h. Cells were washed and then lysed in protein extraction reagent. Lysates were assayed for firefly and Renilla luciferase activity using the dual luciferase assay kit (Promega). To account for differences in transfection efficiency, firefly luciferase activity of each sample was normalized to Renilla luciferase activity.

### Materials

Tannic acid was from Sigma-Aldrich (catalog # 403040). TGF-β was from EMD Millipore (catalog # GF111). Pharmaceutical grade bleomycin was from Teva Pharmaceuticals. Antibodies for Western blotting against SM α-actin (catalog # A5228), β-actin (catalog # A5441), and α-tubulin (catalog # T6074) were from Sigma-Aldrich; against human collagen-1A1 was from Santa Cruz Biotechnology (catalog # sc-28657); against mouse collagen 1 was from EMD Millipore (catalog # 234167); against Smad2 (catalog # L1603) and phospho-Smad2 (catalog # 13804) from Cell Signaling Technology. The SBE4-Luc plasmid was kindly provided by Dr. Bert Vogelstein (The Johns Hopkins University School of Medicine, Baltimore, MD). pRL-TK plasmid was from Promega.

### Statistical analysis

Quantitative data were analyzed by Student’s T-test. Values of *p* < 0.05 were considered statistically significant.

## Results

**Fig. 1:**
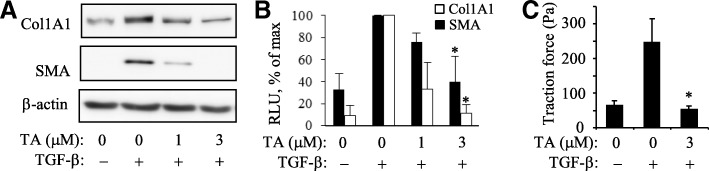
Inhibition of myofibroblast differentiation by tannic acid. **a**, **b**, Inhibition of Collagen-1 and SM-a-actin (SMA) expression by tannic acid (TA) in primary cultures of human lung fibroblasts in response to TGF-β (1 ng/ml, 48 h). Shown are the representative Western blots (**a**) and quantified densitometry (mean ± SD) from at least 3 independent experiments. **c**, Inhibition of cell force generation by tannic acid in response to TGF-β (1 ng/ml, 48 h). Shown are the results of a representative experiment performed in triplicates. *, *p* < 0.05 over TGF-β without TA

Figure 1 shows that tannic acid potently and dose-dependently inhibits TGF-β-induced expression of collagen-1 and smooth muscle α-actin (SMA) without affecting β-actin levels in primary cultured human lung fibroblasts (HLFs) (Fig. 1a, b). Treatment of HLF with TGF-β resulted in a dramatic increase in cell force generation as measured by traction microscopy; and this effect was abolished by pretreatment with tannic acid (Fig. 1c). These data suggest that tannic acid is a powerful inhibitor of myofibroblast differentiation in response to TGF-β in cultured cells.

**Fig. 2:**
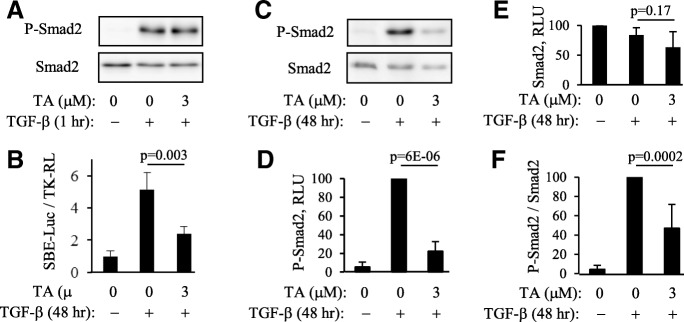
Tannic acid inhibits sustained Smad2 phosphorylation and Smad-dependent gene transcription without affecting initial Smad2 phosphorylation in response to TGF-β. **a**, Pretreatment of HLF with tannic acid (TA) does not affect initial Smad2 phosphorylation in response to TGF-β (1 ng/ml). Shown are the representative results for P-Smad2 and Smad2 Western blotting from one of two independent experiments. **b**, Pretreatment with tannic acid inhibits TGF-β-induced Smad-dependent gene transcription, as assessed using SBE-luciferase reporter (shown are the representative results from one of two independent experiments performed in triplicates). **c**-**f**, Tannic acid inhibits the sustained phosphorylation of Smad2 in response to TGF-β. **c**, Representative P-Smad2 and Smad2 Western blot images; **d**-**f**, Densitometry of P-Smad2 (**d**), Smad2 (**e**), and normalized P-Smad2 / Smad2 (**f**) images. Shown are the means ± SD from at least three independent experiments

TGF-β signals through activation of TGF-β receptors and subsequent phosphorylation and activation of Smad transcription factors driving gene transcription through binding to Smad-binding elements (SBE) on the promoters of target genes. Pretreatment of cells with tannic acid did not affect acute Smad2 phosphorylation in response to TGF-β (Fig. 2a). However, tannic acid significantly inhibited TGF-β-induced Smad-dependent gene transcription within 48 h of treatment, as determined using the SBE-luciferase reporter (Fig. 2b). These data suggest that the antifibrotic effect of tannic acid is independent of the initial TGF-β signaling, but tannic acid still affects the overall Smad-dependent gene transcription in response to TGF-β. We have previously described a critical role of a sustained Smad2 phosphorylation for myofibroblast differentiation [36]. In the present study, we found that tannic acid significantly inhibited sustained Smad2 phosphorylation induced by TGF-β over 48-h time period (Fig. 2c-f), which may explain the effect of tannic acid on myofibroblast differentiation.

**Fig. 3:**
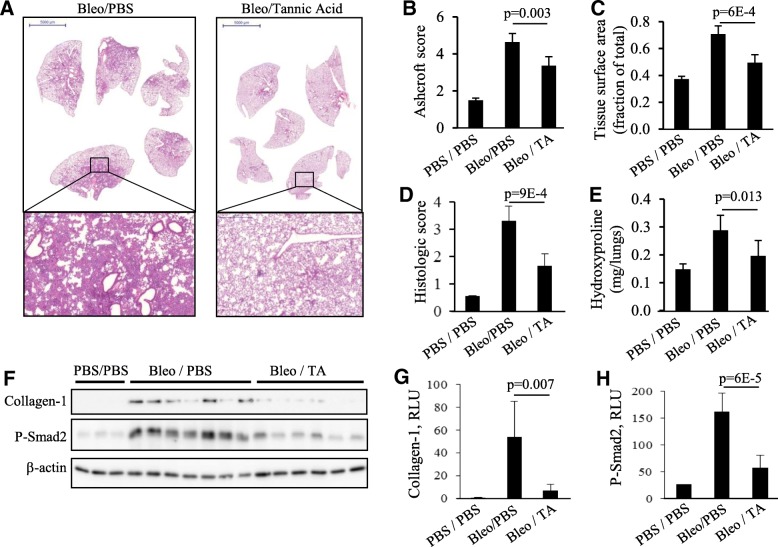
Inhibition of bleomycin-induced pulmonary fibrosis by tannic acid. Tannic acid (TA) treatment (10 mg/kg, i.p., daily) started 7 days after intratracheal administration of bleomycin, and mice were sacrificed on day-21. **a**, representative images of H&E staining of paraffin-embedded lung sections. **b**-**d**, Histologic assessment of **h**&**e**-stained sections for a degree of fibrosis: **b**, Ascroft score; **c**, lung tissue surface area; **d**, histologic score. **e**, hydroxyproline assay of lung extracts for a content of collagen. **f**-**h**, Western blot analysis of lung extracts for a content of Laemmli-buffer-soluble collagen-1, phosphorylated Smad2 (P-Smad2) and β-actin (**f**), and ECL intensity of Collagen-1 (**g**) and P-Smad2 (**h**) blots; mean ± SD

To examine the effect of tannic acid on fibrogenesis in vivo, we used the bleomycin model of pulmonary fibrosis in mice. In this model, intratracheal administration of bleomycin results in an initial injury of alveolar epithelial cells followed by an inflammatory response during the first week, and development of pulmonary fibrosis at two to three weeks post bleomycin administration. To avoid a potential effect of tannic acid on the initial injury and inflammatory response, tannic acid was administered daily beginning on day-7 post bleomycin administration, and the lungs were analyzed on day 21. As shown in Fig. 3, bleomycin treatment resulted in a substantial lung fibrosis, which was significantly attenuated by tannic acid treatment (Fig. 3a). This was quantitatively determined by Ashcroft scoring (Fig. 3b), by measuring lung tissue surface area (Fig. 3c), and by calculating the histologic score (Fig. 3d). Treatment with tannic acid also resulted in a significantly reduced collagen deposition, as assessed biochemically by hydroxyproline assay (Fig. 3e); and in the amounts of Laemmli buffer-soluble Collagen-1, as assessed by Western blotting of lung extracts with collagen-1 antibodies (Fig. 3f, g). Importantly, the bleomycin-treated group showed highly elevated levels of phospho-Smad2, and this was significantly attenuated by tannic acid treatment (Fig. 3f, h). The images of entire Western blots for Collagen-1 and phospho-Smad2 are shown in Additional file 1: Figure S1. Together, these data demonstrate the antifibrotic effect of tannic acid in bleomycin model of pulmonary fibrosis, which is accompanied by inhibition of Smad2 phosphorylation by tannic acid treatment.

## Discussion

In this report, we demonstrate for the first time the powerful anti-fibrotic effect of tannic acid in cultured human lung fibroblasts in vitro and in the bleomycin model of pulmonary fibrosis in vivo. Recent studies have demonstrated the protective effect of tannic acid in a rat model of pressure overload-induced cardiac hypertrophy induced by abdominal aortic banding through a reduction of oxidative stress, inflammation, and fibrosis [27]. It was also shown that tannic acid exerts significant liver-protective effects in mice with CCl_4_-induced liver fibrosis [28]. Our studies show the anti-fibrotic effect of tannic acid in bleomycin model of pulmonary fibrosis (Fig. 3). Importantly, the anti-fibrotic effect in vivo was evident when tannic acid treatment started 7 days post-bleomycin treatment – the time when the initial injury is largely completed. Furthermore, under these treatment conditions, tannic acid significantly inhibited phosphorylation of Smad2 in bleomycin-treated lungs (Fig. 3f, h). It is well established that TGF-β is released during fibrogenesis in bleomycin model of pulmonary fibrosis and contributes to this process [10, 11, 13, 14]. Phosphorylation and nuclear localization of Smad2/3 during lung fibrogenesis in bleomycin model was also shown [33, 37]. Studies have also demonstrated the antifibrotic effect of therapeutic application of other drugs, accompanied by a reduced TGF-β levels and Smad2/3 phosphorylation [33]. However, the mechanisms by which the antifibrotic drugs affect TGF-β/Smad signaling in vivo remain poorly understood.

Our in vitro mechanistic studies suggest that inhibition TGF-β-induced myofibroblast differentiation by tannic acid may be mediated by its effect on a sustained (but not acute) Smad2 phosphorylation, whose critical role in the process of myofibroblast differentiation we recently demonstrated [36]. While our studies were in process, it has been reported that tannic acid inhibits TGF-β-induced epithelial-to-mesenchymal transition in epithelial cells [38]. However, the effective doses used in that study (15–25 μM) were much higher than those in our study (3 μM). In HLFs, tannic acid induced significant cell toxicity at concentrations higher than 10 μM, but not at 3 μM (data not shown). The mentioned above study suggested two mechanisms for the action of tannic acid. First, the authors proposed that inhibition TGF-β receptor-1 (TGFBR1) expression by tannic acid in epithelial cells could be one mechanism [38]. We observed a modest decrease in TGFBR1 expression after 24-h treatment of HLF with 3 μM tannic acid; however, this did not affect the ability of TGF-β to stimulate acute Smad2 phosphorylation (Additional file 2: Figure S2). Second, it was suggested that tannic acid may directly interact with TGF-β and prevent its binding to TGF-β receptors [38]. It was shown that tannic acid, at concentration of 15–25 μM but not at 5 μM, inhibits TGF-β-induced acute Smad phosphorylation in epithelial cells [38]. Given that in our experiments, tannic acid, at concentration of 3 μM that is effective in inhibition of myofibroblast differentiation, had no effect on TGF-β-induced acute Smad2 phosphorylation in HLFs (Fig. 2a), these proposed mechanisms do not explain the anti-fibrotic effect of tannic acid in our study and require further investigation. It would be also important to investigate if the similar effects and mechanisms of regulation of myfibroblast phenotype by tannic acid that we describe in this study would translate to human lung fibroblasts cultured from IPF patients. Finally, the effect of tannic acid on other cells (i.e. alveolar epithelial cells, macrophages and other immune cells) would be important to investigate in the context of pulmonary fibrosis.

## Conclusions

This study demonstrates the anti-fibrotic effect of tannic acid in cultured human lung fibroblasts and in bleomycin model of pulmonary fibrosis. The anti-fibrotic mechanism of tannic acid involved at least in part the regulation of sustained Smad2 phosphorylation in response to TGF-β.

## Additional files

Additional file 1: **Figure S1.** Images of the entire blots for Collagen-1 and P-Smad2 shown in Fig. 3d. (PDF 110 kb)

Additional file 2: **Figure S2.** Effect of tannic acid pretreatment on the acute Smad2 phosphorylation by TGF-β. HLF were pretreated with indicated concentrations of tannic acid (TA) for 1 h or 24 h followed by stimulation with TGF-b for 1 h. Cell lysates were analyzed by Western blotting with antibodies as indicated. (PDF 31 kb)

## Data Availability

The data used and analyzed as well as human lung fibroblast cultures used during this study are available from the corresponding author upon reasonable request.

## Abbreviations

DMEM: Dulbecco’s Modified Eagle’s Medium
HLF: Human lung fibroblasts
HRP: Horseradish peroxidase
IPF: Idiopathic pulmonary fibrosis
PA: Polyacrylamide
Rl: Renilla
SDS: Sodium dodecyl sulphate
SMA: Smooth muscle α-actin
TA: Tannic acid
TGF: Transforming growth factor
TGFBR: TGF-β receptor
TK: Thymidine kinase
