# Supplementary material for: Anti-fibrotic effects of tannic acid through regulation of a sustained TGF-beta receptor signaling
Source: Respir Res. 2019 Jul 29;20:168. doi: 10.1186/s12931-019-1141-8 (PMC6664561; doi:10.1186/s12931-019-1141-8)
Supplement: Supplementary file 1 — Figure S1. Images of the entire blots for Collagen-1 and P-Smad2 shown in Fig. 3d. (PDF 110 kb) [file 12931_2019_1141_MOESM1_ESM.pdf]

A. Image of the entire blot for Collagen-1 shown in figure 3D.

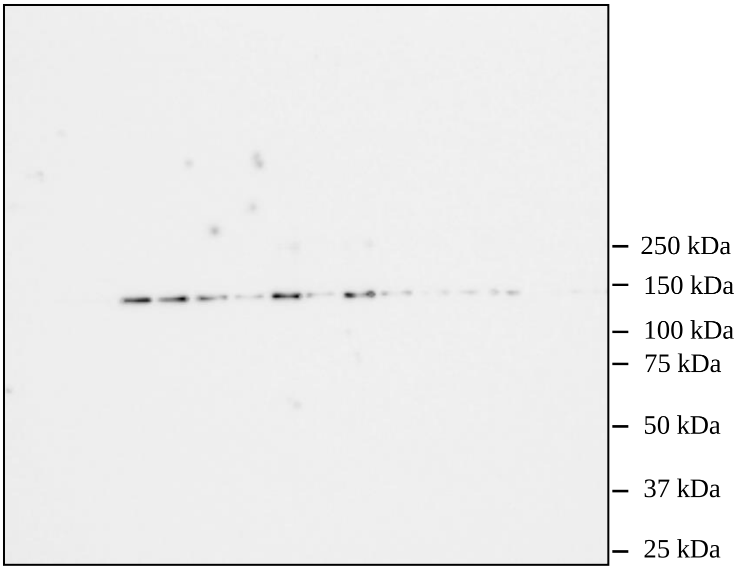

B. Image of the entire blot for P-Smad2 shown in figure 3D.

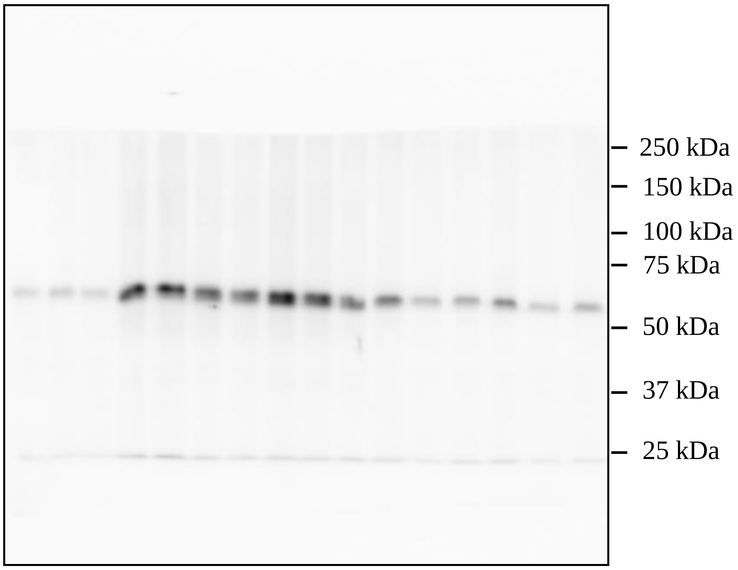

Supplemental Figure S1. Images of the entire blots for Collagen-1 and P-Smad2 shown in figure 3D.
