# Supplementary material for: Anti-fibrotic effects of tannic acid through regulation of a sustained TGF-beta receptor signaling
Source: Respir Res. 2019 Jul 29;20:168. doi: 10.1186/s12931-019-1141-8 (PMC6664561; doi:10.1186/s12931-019-1141-8)
Supplement: Supplementary file 2 — Figure S2. Effect of tannic acid pretreatment on the acute Smad2 phosphorylation by TGF-β. HLF were pretreated with indicated concentrations of tannic acid (TA) for 1 h or 24 h followed by stimulation with TGF-b for 1 h. Cell lysates were analyzed by Western blotting with antibodies as indicated. (PDF 31 kb) [file 12931_2019_1141_MOESM2_ESM.pdf]

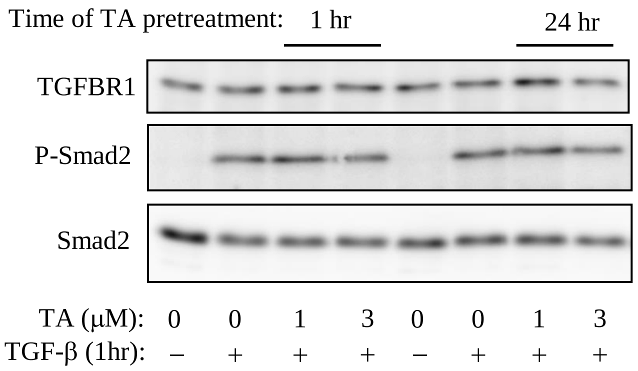

Supplemental Figure S2. Effect of tannic acid pretreatment on the acute Smad2 phosphorylation by TGF-β. HLF were pretreated with indicated concentrations of tannic acid (TA) for 1hr or 24 hr followed by stimulation with TGF-β for 1 hour. Cell lysates were analyzed by Western blotting with antibodies as indicated.
